# Microbiome ethics, guiding principles for microbiome research, use and knowledge management

**DOI:** 10.1186/s40793-022-00444-y

**Published:** 2022-09-30

**Authors:** Lene Lange, Gabriele Berg, Tomislav Cernava, Marie-Christine Champomier-Vergès, Trevor Charles, Luca Cocolin, Paul Cotter, Kathleen D’Hondt, Tanja Kostic, Emmanuelle Maguin, Thulani Makhalanyane, Annelein Meisner, Matthew Ryan, George Seghal Kiran, Rafael Soares de Souza, Yolanda Sanz, Michael Schloter, Hauke Smidt, Steve Wakelin, Angela Sessitsch

**Affiliations:** 1 LL-BioEconomy, Valby, Copenhagen, Denmark; 2 Graz University of Technology, Graz, Austria; 3 INRAE, AgroParisTech, Micalis Institute, Université Paris-Saclay, Jouy-en-Josas, France; 4 University of Waterloo, Waterloo, Canada; 5 University of Turin, Grugliasco, Italy; 6 Teagasc Food Research Centre, Moorepark, APC Microbiome Ireland and VistaMilk, Cork, Ireland; 7 Department of Economy, Science and Innovation, Flemish Government, Brussels, Belgium; 8 AIT Austrian Institute of Technology GmbH, Tulln, Austria; 9 University of Pretoria, Pretoria, South Africa; 10 Wageningen Research, Wageningen University & Research, Wageningen, The Netherlands; 11 Genetic Resources Collection, CABI, Egham, UK; 12 Pondicherry University, Puducherry, India; 13 State University of Campinas, Campinas, Brazil; 14 Institute of Agrochemistry and Food Technology- Spanish National Research Council (IATA-CSIC), Valencia, Spain; 15 Helmholtz Zentrum München, Oberschleissheim, Germany; 16 Laboratory of Microbiology, Wageningen University & Research, Wageningen, The Netherlands; 17 Scion, Christchurch, New Zealand

**Keywords:** Microbiome, Ethics, Planetary health, FAIR principles, Global common heritage, FAO International Treaty

## Abstract

The overarching biological impact of microbiomes on their hosts, and more generally their environment, reflects the co-evolution of a mutualistic symbiosis, generating fitness for both. Knowledge of microbiomes, their systemic role, interactions, and impact grows exponentially. When a research field of importance for planetary health evolves so rapidly, it is essential to consider it from an ethical holistic perspective. However, to date, the topic of microbiome ethics has received relatively little attention considering its importance. Here, ethical analysis of microbiome research, innovation, use, and potential impact is structured around the four cornerstone principles of ethics: Do Good; Don’t Harm; Respect; Act Justly. This simple, but not simplistic approach allows ethical issues to be communicative and operational. The essence of the paper is captured in a set of eleven microbiome ethics recommendations, e.g., proposing gut microbiome status as common global heritage, similar to the internationally agreed status of major food crops.

## Introduction

**Fig. 1:**
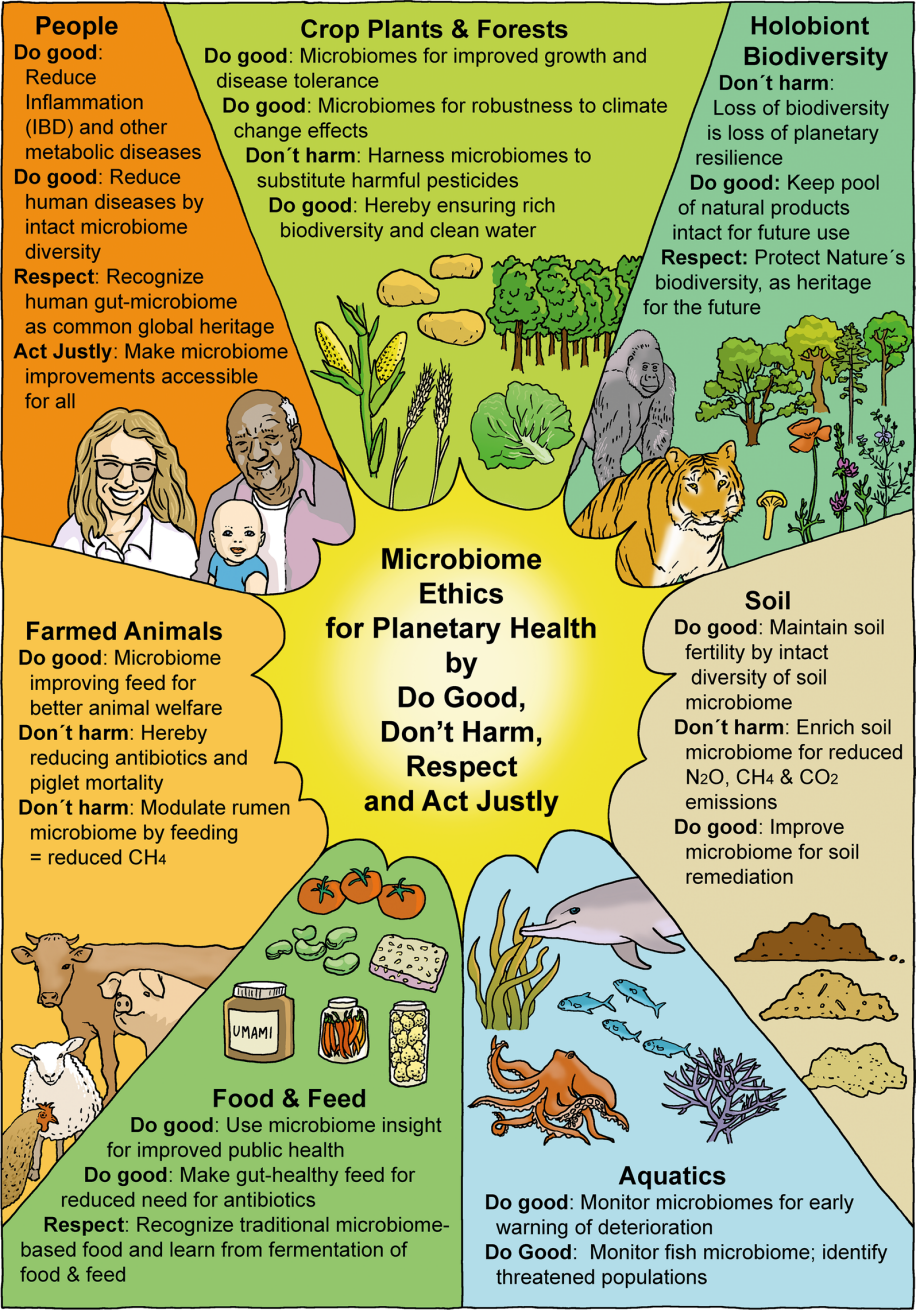
In centre, the four core principles of ethics (Do Good, Don´t Harm, Respect, Act Justly), applied here for analysis of microbiome ethics, issues, and dilemmas; illustrated by selected examples within the major types of microbiomes, Crop Plants and Forests, Holobiont Biodiversity, Soil, Aquatics, Food & Feed, Farmed Animals and People

A microbiome is a microbial community, comprising viruses, bacteria, archaea, unicellular eukaryotes and fungi, as well as the activities of these organisms. Microbiomes are characteristic for a specific habitat, and closely connected to their host or environment [1]. Microbiomes are highly complex and occur in any environment comprising e.g., humans, animals, plants, insects, algae, soils, water systems or air. Microbiomes have crucial roles in maintaining life on Earth and have been identified as a key component of the health of all Eukaryotes, including humans. The biological impact of microbiomes on their hosts reflects the co-evolution of a mutualistic symbiosis, generating fitness for both. Knowledge on microbiomes, their systemic role, types of interactions and their 'theatre of activities' grows exponentially [1, 2]. When new knowledge and technologies of importance for planetary health and well-being are evolving so rapidly, it is essential to consider and analyse ethical principles to guide the rapidly growing field of microbiome research, use and knowledge management. Conducive to such analysis is the planetary health approach [3] and the planetary boundary concept, resulting in the need for careful assessment of both opportunities and possible negative health or environmental impacts of microbiome-based innovations [1]. To date, relatively few papers have addressed ethics applied to microbiome research [4–6]. As an overarching principle, we structure our evaluation around four cornerstone principles of ethics: Do Good; Don’t Harm; Respect; Act Justly. This is a simple, but not simplistic approach, which enables the analysis of ethics-related issues to be communicative and operational. The background for our choice of these four over-arching guiding principles for ethical analysis of microbiome ethics is based on the experience of the authors: The use of a steadily increasing number of biobased technologies in industry as well as expanding the scope of annual reporting of biotech business (from only financial to include also environmental and social reporting) created a need for a clear and systematic analytical approach also to ethical standards. Bioethics analysis of e.g., industrial biotech uses of microbes and microbial enzymes as well as genetically modified (GM) microorganisms (under biologically contained conditions) was found to be both comprehensive and intuitively understandable also for non-specialists, when elucidated by addressing these four corner stone concepts: Do Good, Don’t Harm, Respect and Act Justly. Based on this experience we chose to use this analytical approach also for the new bioethics field, the microbiome ethics (see also Fig. 1, where the use of the four ethical core principles is exemplified). Further, for data handling within microbiome research, it is important to follow the FAIR principles—findability, accessibility, interoperability, and reusability, which have been agreed on in 2016 [7].

## Microbiome impact is essential

The urgent need to include ethical perspectives and analysis (identifying issues and dilemmas) in microbiome research stems from the fact, that new knowledge has led to remarkable insights into the fundamental impact that microbiomes have, not just on human health and the entire food system (soil, plants, animals), but also on planetary health (see overview of microbiome ethics issues, Fig. 1). Microbiome research has generated valuable insights in different food system microbiomes, including the microbiomes of soil, farmed animals and terrestrial and aquatic ecosystems [8–15], underpinning immense impact on plant growth, yield, animal welfare, soil health and biodiversity. The prominent impact of the human gut microbiome is now backed by a rapidly growing evidence-base, relating to: (1) physiology and functions e.g., development, child growth [16], ageing [17], preterm birth [18], digestion and response to diet [19], immunity and nervous system function [20]; and (2) various diseases e.g., metabolic syndrome [21], obesity [22], diabetes type 2 [23], inflammatory bowel disease [24], mental disorders [25] and cancer immunotherapy [26].

The exponential growth in available microbiome data, combined with significant advances in “-omics” technologies such as genomics, transcriptomics, proteomics, metabolomics, and culturomics [27], has led to ground-breaking advances in our understanding of the potential of microbiome-based innovations to enhance the productivity of food systems and beyond. We now know, that it is possible to modulate and improve both the organismal diversity and the function of microbiomes. Similarly, the influence of microbiomes on farm animals goes beyond productivity (meat, milk, egg), as it impacts both animal health and welfare. Further, skin and mucosa-associated microbiomes (lung, gut) are essential for safe-guarding humans and animals from invading pathogens. A prominent problem in industrial meat production is an inflammatory gut, leading to premature and painful death of millions of piglets. The measure often used against animal inflammatory gut is flock-treatment with antibiotics, when one or few animals are sick. This practice inadvertently leads to higher risk of developing antimicrobial resistance (AMR) and consequently increases the risk of AMR-caused pandemics of non-curable infectious diseases in humans and animals. As microbiomes can have a major impact on human and animal health, and measures to improve microbiomes are available, this issue accentuates the need for awareness of ethical dilemmas, e.g., economic gains versus animal welfare or antibiotic use versus enhanced risk of AMR-associated pandemics.

A recent paper [4] hypothesizes that the selection for metabolically weight-gaining animals for efficient industrial meat production inadvertently selects for gut microbiota favouring obesity. Also, bakers’ hand microbiomes are related to the composition of their sourdough and vice versa [28], evidencing the interconnectivity of microbiomes in animals, humans and the environment. Further, the impact of host genetics, whether plant, animal or human, on their microbiome composition and function is far from being elucidated. Notably, more research is needed as informed basis for new health-improving microbiome-based applications and promoting new products without evidence-base may be misleading or even harmful. It leaves a strong plea to address microbiome research in a planetary approach, embracing different types of microbiomes, and assessing the impact of intervention on one type of microbiome with a systems approach [2].

Microbiome research has in the last years been driven by (meta-)genome sequencing and annotation of microbiomes [1]. However, metagenomic analysis may often not give the full picture. Some of the DNA identified could be from dead or not actively growing microorganisms [29], some important taxa could be below the detection level, though still of relevance for ecosystem function [30], and functional genes identified are not always expressed. Notably, microbiome research is now moving towards taking a more comprehensive analytical approach to include and integrate advanced “culturomics”, functional annotation, meta-transcriptomics, meta-proteomics and meta-metabolomics [31]. Further, advanced microbiome analyses should embrace not only prokaryotes but also micro-eukaryotes (protozoa and fungi) and viruses (including bacteriophages). Overall, a more comprehensive understanding of microbiome complexity is needed to obtain better insights into the roles and functions of microbiomes, thereby leading also to an improved basis to address ethics-related aspects. Here, data availability plays an important role, to make the reuse of data possible for new forms of analysis but also for an integrated evaluation of data, as suggested by the FAIR principles [7]. Also, controlled storage of samples, from where the “omics” data were derived and accessibility to additional data will be of crucial importance to improve our understanding on the complex interactions of microbiota with themselves, their host, and their abiotic environment. Therefore, the role of biobanks and microbial culture collections needs to be further strengthened [32].

## Microbiome ethics and bioethics

Microbiome ethics relates to several recognized fields of ethics, including bioethics, environmental ethics and food ethics. Animal welfare at large is supported by bioethics, and by microbiome ethics, as improved microbiomes may reduce animal suffering. Biodiversity and conservation biology are a focus of environmental ethics, but also of microbiome ethics, as e.g., soil/plant microbiome improvements can reduce the need for chemical pesticides or fertilizers (see Fig. 1). In food ethics, the promotion of foods, high in fat, sugar and salt is of ethical concern, as such foods are known to be unhealthy and disturb the gut microbiome. Microbiome-derived interventions to improve a distorted gut microbiome are thus relevant to microbiome ethics. Furthermore, human microbiome preventive and therapeutic uses (e.g., faecal microbiome transplants) require bioethical considerations [33]. Another emerging topic of ethical relevance is where anthropogenic-incurred wildlife biodiversity loss has reached a stage, where microbiome-targeted interventions provide the only solution [34].

## Addressing the ethical aspects

Ethical aspects related to microbiome research and microbiome knowledge-derived use has been most extensively considered in human microbiome research and medicine. The approach here was to develop rules, standards, and procedures for legitimate access to materials (to be used for microbiome research) and on data handling and use (informed consent, data sharing and use of results). Considering a broader ethical perspective on microbiome research, (including use and societal impact) is timely, as the basis has been generated to develop new types of microbiome-based products, targeting environmental, plant, animal and human health. These products typically include feed additives, food ingredients/supplements, drugs, soil improvers, or plant protection products. In the near future, this product range and range of possible applications is expected to broaden significantly, including new products for optimizing soil health, plant resilience or nutrient-efficiency; applications rescuing endangered species; or products mitigating climate change, e.g., by insightful modulation of the rumen microbiome, leading to reduced methane emission. Therefore, a significant increase in patenting, regulatory approval, labelling and application of microbiome-based innovations, including their associated ethical issues, must be anticipated. The need is now; time is short [35].

## The burning questions

How do we ensure that future, potentially disruptive, microbiome knowledge-based and microbiome-derived treatments in the health and food system and beyond are being developed globally, for the benefit of all, supporting environmental, dietary, and ethnic diversity? While we have developed a system of informed consent for use of organs, tissues, and other bio-samples, the handling of microbiomes remains largely unsettled. Do we have the ideal system in place for microbial strains isolated from human individuals (or their faeces)? Are we in gear with regard to establishing a similar system, ensuring that respect for human integrity also cover its microbiota? Over decades the UN system has made important consensus efforts [36] to protect the sovereign rights of the country of origin with respect to biodiversity, by requiring prior informed consent under mutually agreed terms [37]. But what about microbiome-derived microbial species for human or animal use? Should it be permissible to issue use-patents of such signature microbes, hereby restricting their use for the benefit of man and the environment to individual companies? Notably, the global society has developed an international treaty defining that the 64 major food crops of the world do not belong to a specific country, breeding company or biotech enterprise [38]. They belong to humanity. They comprise a cornerstone of our global common heritage and is central to our shared planetary future. Perhaps the keystone species of the human gut microbiome, of all ethnicities and all types of dietary habits, should also be considered common heritage? Similarly, ethical considerations have to be discussed with respect to microbiomes and species, associated with production animals and other parts of the global food system.

## The cornerstone concepts in ethics

The four important cornerstone concepts of ethics, i.e., Do Good; Don`t Harm; Respect; and Act Justly, were selected to facilitate and structure the analysis of ethical perspectives within microbiome research, innovations and microbiome-derived interventions and applications. We believe this reductionistic approach, which is simple, but not simplistic, can pave the way for analysis of ethical dilemmas, hereby opening for more responsible research and innovation, leading to well thought-through knowledge management and use, for the benefit of man, animals, plants, soil, food, biodiversity, and planetary health at large.

### Do good

To do good, knowledge derived from microbiome research should, to the widest extent, be placed in the public domain. Public research in this field should be prioritized and public–private collaboration encouraged, to facilitate beneficial uses being generally accessible. When an area is universally important, e.g., for planetary and environmental health, there is a moral obligation to make dedicated efforts to share and spread such knowledge and to make technologies and products accessible for all. We should aim at empowering all countries and regions to generate and use such knowledge, as a basis for developing affordable local products and practices for strengthening microbiomes, in all parts of the body and in all ecosystems, sustaining and enhancing biodiversity. This would also contribute to protecting biodiversity (e.g., by reducing pesticide use), ensuring food security and improve nutrition. For example, efforts should enable the introduction of microbiome-based gut health food and feed products (viz. prebiotic ingredients, probiotic cultures and live biotherapeutics; anti-inflammatory/immunomodulating compounds), to improve human health and animal welfare, and enabling significant reduction of antibiotic usage, lowering the threat of pandemic disease emergence from AMR microorganisms.

### Don’t harm

Introducing clear and precise quality and safety testing principles for new microbiome knowledge-based applications (inventions, interventions, and products) is necessary to avoid any risk of harmful side-effects. Clear regulatory approval guidelines are urgently required for the timely development of novel, useful and safe commercial products. The development of principles and guidelines for patenting microbiome evidence-based inventions is needed to avoid obstacles are being created for the wider, generic use of traditional microbiome knowledge for human, animal, plant, soil, and planetary health. More specifically, new generic, non-proprietary and safe practices, and products, locally as well as globally, should not be blocked for use by process-, product-, or use patents. Further, concerted efforts are needed to avoid potential harm, if new essential products (for health and well-being) are delayed in regulatory assessment. Benefits of novel microbiome insight should reach all groups of citizens and all parts of the world to avoid doing harm due to lack of information; thus, priority should be given to dissemination, communication, technology transfer and training programs about microbiome-based research, results, and innovations. If not done, we de facto block the overarching beneficial opportunities of microbiome insight from reaching all parts of the world. This is in itself is “doing harm”.

### Respect

Respect should be given to regiments of treatments and food practices (indigenous or traditional, local or regional) that are in use and proven historically safe, even if their mode-of-action has not been elucidated. In particular, respect should be given to practices, using locally derived dietary fibres and other traditional products that are promoting improved gut health, digestion or metabolism, and may be part of traditional medicine or traditional food, respecting traditions with no health risks for consumers [39]. Similarly, respect should be given to uses of microbes and microbially-derived products such as e.g., microbial biocontrol agents (against diseases and pests) and soil improvement products [40]. Respect should be also paid to innovative, knowledge-based, safe entrepreneurial efforts, by allowing appropriate and responsible technology protection and providing an enabling, supportive, and efficient regulatory approval process. Lastly, the right of the individual to choose, which microbiome-derived products to use, take or consume, should be shown respect by clear labelling, informing e.g., about microbes used, being wild-type, CRISPR-Cas or GM-constructs, hereby allowing for user´s informed choice.

### Act justly

It is neither just nor fair when knowledge that is essential for health and well-being of the biosphere, is not accessible. Targeted, focused, timely and enduring efforts should be vested into global knowledge-sharing and dissemination of research methodologies and microbiome impacting know-how in all continents. Indigenous people´s rights should be considered in a fair and just manner, ensuring that no patents create obstacles for beneficial effects derived from indigenous types of microbiomes, as whole consortia or in part. This would assure that knowledge-based products for improved microbiomes can be accessible for all types of dietary and ethnicity populations. Basically, products for improving the food systems, including gut ecosystem and host health, such as probiotics, prebiotics, anti-inflammatory immunomodulating compounds, can be produced at low cost from food-processing side-streams, leading to (fair and just) affordable end-user prices, providing for social inclusiveness in planetary health. Microbiome-based warning systems should be developed, providing short-cuts to capturing of early signals of upcoming threats to public health of humans, animals or plants, soils, or aquatic environments. These should be shared freely, internationally, timely and openly. Monitoring of soil health, protecting biodiversity and planetary health (including climate change and biodiversity loss) through microbiome analysis is part of the “Act Justly” concept, as degrading planetary health unavoidably damages society in a socially biased manner.

### Eleven key messages based on the ethical analysis

The essence of the paper is summarized as a set of eleven guiding principles for microbiome research, based on ethical analysis, all being of global relevance (Text Box 1).

**Box 1:** Eleven Guiding Principles for microbiome research, based on ethical analysis

1. Establish common ethical codes-of-conduct for microbiome applications, considering whole ecosystems, keeping in mind the planetary health concept; stimulate and strengthen public research and knowledge sharing; place knowledge in the public domain; increase awareness
2. Consider the human gut microbiome as global common heritage; seen as a continuum to the FAO International Treaty on plant genetic resources for food and agriculture, where 64 major food crops hold this status
3. Facilitate the deposition of microbiome sequences as open-source, accessible for all; establish a sequence database of microbial diversity for the health of man, animal, and plants; for improved resilience to climate change challenges and pandemics
4. Provide open access microbiome-relevant culture collections as a source of not-patented, safe-to-use, key microbiome species/specimens/consortia, available for microbiome-improvement of soils and for strengthening resilience in humans, crops, trees, and animals (including wildlife)
5. Stimulate international scientific collaboration within microbiome research, technologies, innovations and uses, including all parts of the world, public and private, for the benefit of health and well-being of humanity; contributing to planetary health
6. Stimulate public–private collaboration within microbiome research and innovation, enabling products available and accessible for improved microbiome diversity within food ecosystems. IP-protection should be by claiming specific inventive steps only; no broader microbiome use claims
7. Use microbiome insight for gut health-promoting products also where most needed. Climate change threatens food security in drought-stricken Sub-Saharan Africa; affordable food, made from local residues or processing side-streams can in a fair and just manner improve public health
8. Stimulate microbiome research with a holistic approach, spanning across different microbiome systems; microbiome research silos delay conceptually new microbiome insight, delaying potentially life-saving innovations and use
9. Stimulate microbiome research, elucidating conducive conditions for the serious, widespread global obesity and malnutrition pandemics as well as for solutions to support sustainable and responsible agricultural production
10. Stimulate soil–plant microbiome research, for increased carbon sequestration and N and P (re)cycling, and for monitoring biodiversity (at species and habitat level), identifying climate change-induced changes in microbiome composition and function
11. Prioritize microbiome research for early warning of pandemics

## Future perspectives

In the coming years, microbiome research, insight and applications will impact many more areas of use. In a planetary health perspective, considering the diversity of all organisms in various connected habitats, a holistic approach to microbiome research can bring forward improved understanding of how climate change modifies the biosphere [2]. Such system insight can be used to design new microbiome-based approaches to monitor detrimental effects on biodiversity/biosphere before these effects are apparent. To unlock such potential, new methodological developments in microbiome research must take place, most importantly by including all types of organisms in the studies, optimizing functional annotation and optimize identification of metabolites in the microbiome secretome [41]; and not the least by improved technologies for storing intact samples in biobanks or culture collections.

Microbiomes are the closest and most integrated interactions people, animal, plants, insects, macrofungi and algae have with microorganisms; and microbiomes are the first line of defence against pathogens, viruses, bacteria, and fungi as well as other possible harmful environmental exposures (e.g., chemicals). Furthermore, monitoring the biological effects on the soil microbiome may be an important enabling strategy to monitor climate change-derived (and endangering) emissions from for instance thawing permafrost. Forward-looking scientists recently elucidated opportunities, risks and ethics of large-scale microbiome research in a subways & urban biomass project [5], sending a message: Microbiomes are all around us and in us, therefore public engagement in understanding microbiome function and role is imperative.

In large global research programs, microbiome research is generally not given high priority, its complexity is an obstacle for communicating its importance. However, microbiome research is relevant and important for many of the topics prioritized: Food quality, productive and sustainable agriculture, forestry, food safety and food security, circular bio-based economy, reducing waste, upgrading residues/side-streams, protecting soil, improving nutrition, green and healthy-eating, reducing AMR, drug discovery, stopping biodiversity loss and ensuring water quality [42]. Notably, most of these are central tenants of the UN Sustainable Development Goals. Timely and responsible action should ensure, that microbiome studies are integrated in all these areas, and that microbiome ethics, identifying issues and dilemmas, becomes an integrated part of sustainability assessment. Microbiome research and innovation can also contribute to global preparedness for upcoming pandemics. Microbiome-based monitoring may provide essential early warning signals e.g., in case of occurrence and spread of antibiotic resistance among serious infectious pathogens. In general, improved microbiome diversity in humans, animals, plants, and the environment, can add to robustness and resilience. The microbiome ethics-based guidelines (Box 1) send a clear signal: when you have the know-how and safe microbiome applications (with documented beneficial effects) are available and in line with the four principles—Do Good; Don’t Harm; Respect; Act Justly—you have an obligation to act and share.

## Concluding manifesto

We raise questions relevant for microbiome research, innovation, and applications, because it is essential and urgent to have common and open discussions about microbiome ethics. A wide circle of microbiome experts of the European and international microbiome research community was included in writing the paper. We took the bold step to initiate a broader discussion of essential questions. A discussion, founded and structured based on concepts, built along with civilization, grounded in the humanities and shaped by cultures, refined scientifically in the ethics field of philosophy. We realize that we are not specialists in the ethics field of philosophy. However, insight into the field of microbiome research is a necessity to start the discussion. This is the reason why this paper was written; as part of the MicrobiomeSupport project, funded and initiated under the EU Horizon2020 program, including co-authors from 14 countries, across five continents.

## Data Availability

Not applicable.

## Abbreviations

AMR: Antimicrobial resistance
DNA: Deoxyribonucleic acid
UN: United Nations
CRISPR-Cas: Clustered regularly interspaced short palindromic repeats and CRISPR-associated protein
GM: Genetically modified
